# Influence of Teeth Preparation Finishing on the Adaptation of Lithium Disilicate Crowns

**DOI:** 10.1155/2017/2078526

**Published:** 2017-03-23

**Authors:** Bruna Salamoni Sinhori, Mauro Amaral Caldeira de Andrada, Guilherme Carpena Lopes, Sylvio Monteiro Junior, Luiz Narciso Baratieri

**Affiliations:** Federal University of Santa Catarina, Florianopolis, SC, Brazil

## Abstract

The polishing step of teeth preparations for crowns is a step often performed, so that there is an increased time during the clinical procedure. The aim of this study is to evaluate the marginal and internal adaptation of all-ceramic CAD/CAM lithium disilicate crowns in polished preparations for crown and nonpolished preparations for crowns. For this purpose, 20 first molars were selected, which were divided into two groups (*n* = 10) G1, teeth that received surface roughening similar to preparation without polishing, and G2 (control), polished preparations. After the preparations were completed the teeth were scanned (Cerec Bluecam, Sirona, Bensheim, Germany), and the crowns were designed and machined using CAD/CAM technology (Sirona, Bensheim, Germany). The adaptation of the pieces was evaluated using polyvinyl siloxane replicas and stereomicroscope photographs with 70x magnifications. The normality test indicated a nonnormal result, so a Man–Whitney nonparametric test was performed. One out of the 24 measured regions showed a statistically significant difference (*p* = 0.0494). With this study it can be concluded that crowns fabricated by CAD/CAM technology performed on unpolished preparations are not influenced by the internal marginal adaptation and the ceramic part and are different from polished preparations.

## 1. Introduction

Several ceramic systems and manufacturing processes were introduced to the dental market as a consequence of highly aesthetically demanding patients [1, 2]. From the ceramic injection using the lost wax technique to the industrially prefabricated ceramic milling machines developed for CAD/CAM systems, dentistry evolved in the search for alternatives to conventional ceramic layering techniques [3–8]. CAD/CAM systems are mostly used in conjunction with ceramic materials such as lithium disilicate (IPS e.max CAD, Ivoclar Vivadent) [4]. Lithium disilicate is a restorative ceramic material that combines high flexural strength with outstanding aesthetics. Restorations with such material may be manufactured with CAD/CAM system by using prefabricated blocks or by the lost wax technique [9, 10]. Marginal adaptation is a very important aspect when it comes to indirect restorations, because no matter how small the maladaptation is, it will cause small marginal gaps/openings leading to plaque accumulation, production of sulcular fluid, possibility of bone loss and periodontal disease development at the site, infiltration, and recurrent caries [11–13].

The high demand for aesthetic dental restorations made ceramic crowns popular. Three important factors must be emphasized when addressing ceramics: marginal adaptation, internal adaptation, and strength of the material; therefore all ceramic materials used for restorations and crowns must meet these three factors in order to meet the clinical requirement and prolong clinical lifetime [14–18]. The lack of marginal and internal adaptation of ceramic crowns can affect the longevity of indirect dental restorations [19–22]. Faulty restoration margins increase plaque retention, which can cause traumatic gingival irritation and/or short-term decay [23–25].

Adequate internal adaptation is especially important for placement of ceramic restorations because it results in homogeneous distribution of masticatory loads under stress [16, 17, 26–28]. And when the ceramic restoration is well adapted chances of infiltration to occur in the margins of the tooth-restoration assembly are reduced.

The relevance of this research is given by the interest to evaluate whether the polishing for crown preparations is truly necessary for the ceramic restoration to be well adapted, in addition to observe the CAD/CAM system behavior while acquiring images with different degrees of polishing. This study evaluated whether finishing of crown preparations influences the marginal and internal adaptation of lithium disilicate-based ceramic crowns fabricated with the Cerec system. The evaluation was made by a measurement technique at the tooth/ceramic interface with silicone replicas and evaluated through photographs with stereomicroscope under 70x magnification.

Therefore the aim of this study was to evaluate in vitro whether the polishing of crown preparations influences the internal and marginal adaptation of lithium disilicate ceramic crowns fabricated using CAD/CAM technology. The null hypothesis was that the polishing preparation would not influence the internal and marginal adaptation.

## 2. Materials and Methods

For the development of the research, a plastic manikin was used (P-Oclusal), in which the maxillary left first molar teeth was the focus of the study. Twenty premade plastic teeth element number 26, the brand P-Oclusal, with full-crown preparations, were used. The selected teeth were fixed in the plastic manikin, in the position correspondent to the tooth #26. Each tooth was included for the establishment of the procedure according to the group. This procedure was intended to facilitate the subsequent stages of the research.

(i) Cavity preparation: the artificial teeth with full-crown preparations were divided into 2 groups. 

*Group 1*. Preparation shipped from the factory was roughened with ground particle size diamond bur (#3131, KG Sorensen, Brazil), simulating preparations without finishing and polishing steps. 

**Figure 1:**
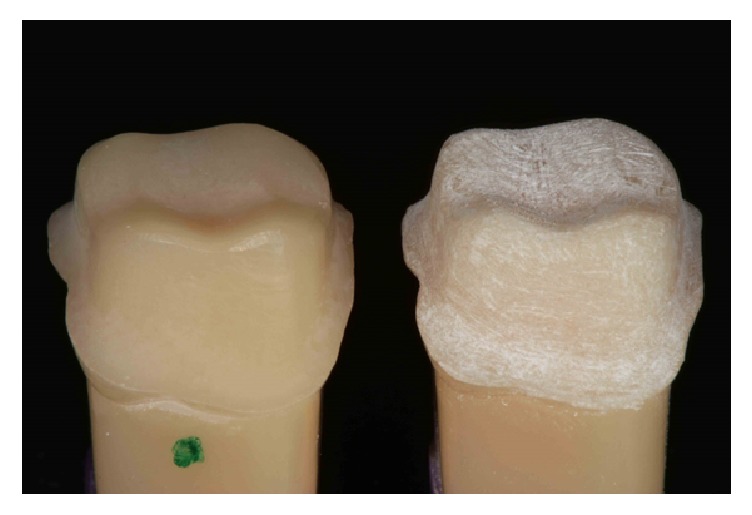
Tooth preparation of Group 2 and Group 1.

*Group 2 (Control)*. Preparation shipped from the factory has not been modified. A preparation was simulated with diamond bur in decreased sequence of particle size, fine (#3131F, KG Sorensen), and extra-fine (#3131FF, KG Sorensen), following the steps of finishing and polishing. Upon completion of the preparations step, the teeth were cleaned with compressed air-water to remove any debris that remained on the surface (Figure 1). 

(ii) The ceramic crowns restorations were fabricated by a CAD/CAM system (Cerec AC, Sirona Dental Systems). Each preparation was covered with titanium dioxide spray (Cerec OptiSpray, Sirona Dental Systems, Batch #2013140338) and scanned (Bluecam, Cerec AC). To standardize the contour anatomy and as the original restoration design was not changed, only “Position,” “Add,” and “Smooth” tools of the software (InLab 4.2, Sirona) were used in the free surfaces to ensure the correct thickness of all regions of the preparation and optimal adaptation. The following parameters were used: Biogeneric Individual, film thickness: 60 *μ*m; occlusal compensation: −300 *μ*m; proximal contact strength: 25 *μ*m; occlusal contact strength: −150 *μ*m; and margin thickness: 0 *μ*m; and Consider Instrument Geometry was set to OFF. Lithium disilicate CAD/CAM ceramic blocks were used for making the crowns (e.max CAD HT, shade B3, size C14, Ivoclar Vivadent). Since these blocks are precrystallized after the milling completed, they were led to a furnace at 840°C for 20 to 25 minutes according to manufacturer's recommendations.

Following the fabrication of the ceramic restorations, began the stage of evaluation of marginal adaptation. The twenty ceramic restorations were divided into two groups according to the type of polishing and finishing of preparations. The marginal adaptation evaluation was performed in all aspects (buccal, mesial, palatal, and distal). The seating of the ceramic restorations was performed by the same calibrated researcher (BSS), by finger pressure. Then, the tooth-restoration interface was then taken to an optical microscope with 70x magnification, where snapshots of the interfaces were taken. No mismatches of the restorations were observed, so the need for internal adjustments was discarded.

The plastic teeth previously divided into two groups and evaluated for marginal adaptation were fixed to a device in order to assist the impression steps with silicone film. To facilitate the apprehension of the teeth to carry out the subsequent stages of evaluation of the internal fit of the preparation, the root portion of each tooth was fixed into a plastic ring (2.0 cm diameter, 2.5 cm height). A polyvinyl siloxane (PVS) impression material was inserted inside the ring (Virtual Putty, Ivoclar Vivadent). The teeth received demarcations on the buccal, mesial, distal, and palatal aspects of the root, 2 mm below the preparation, to standardize the fixation of the specimens for all groups. 

(iii) Internal fit evaluation: for the evaluation of internal fit, the cementation of crowns on their respective teeth was performed with a PVS material. Subsequently, the thickness of this silicone film was measured in 24 different sites for each crown. A PVS material for occlusal registration was used (Flexitime Correct Flow, Heraeus Kulzer), according to manufacturer's guidelines. Immediately, the silicone was injected into the ceramic crown and seated with finger pressure on its respective tooth. The crown/silicone/tooth assembly was fixed in the metallic device, and a constant load of 1 kg was applied for 2:30 minutes, as recommended by the manufacturer.

After the polymerization time, the silicone overflowed was cut with a #15 scalpel. The scalpel blade was replaced by a new one every 3 crowns, to avoid tearing of the PVS material. Then the assembly was removed from the device and the crown was carefully removed from the tooth. Each film was evaluated for possible defects, such as lack of material or even rupture during its removal from the crown. In case of deficiencies, the process was repeated until a silicone film without defects was obtained.

For capture and stabilization of the PVS material film, a PVS material with contrasting color (Flexitime Correct Flow, Heraeus Kulzer) to that of the film used for cementation was selected. Therefore, a low viscosity PVS impression material (Virtual, Light Body Regular Set, Ivoclar Vivadent) was selected for the stabilization of the film (yellow shade), different from the PVS material used for measurement of the internal fit (green). This contrast facilitates the subsequent measurement of the film thickness. A low viscosity addition PVS material (Virtual Light Body Regular Set, Ivoclar Vivadent) was injected on the Flexitime film, with the help of an automixture syringe, starting from the occlusal aspect. With rotary motion, the coverage of the whole film was performed. Simultaneously, an additional portion of the silicone was discharged inside a plastic cylinder, and this was settled on the plastic part with the tooth.

**Figure 2:**
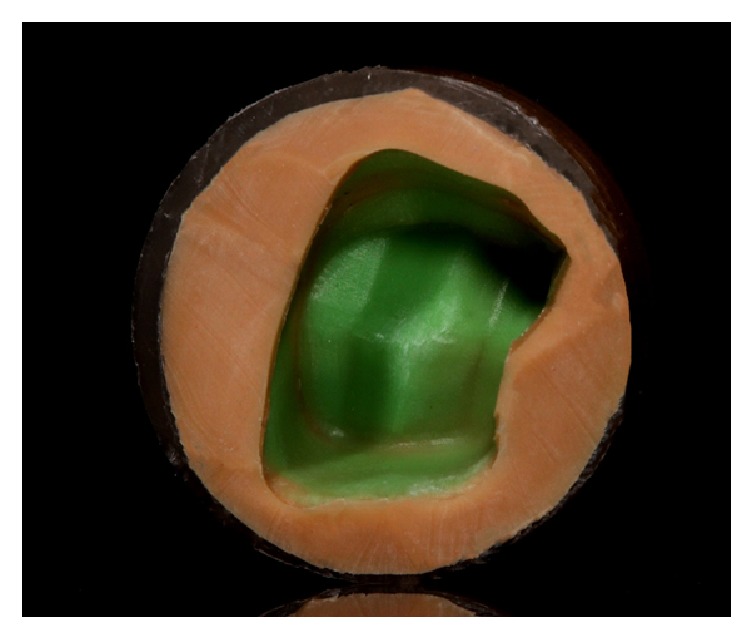
Film capture with light silicone of another color.

The device seated on the tooth preparation was stabilized until the setting of the material occurred, as recommended by the manufacturer, that is, 4:30 minutes. After polymerization, excess silicone was removed, and the silicon portion Light Body Regular Set was identified by letters for the mesial, distal, buccal, and palatal surfaces. The groups were identified by a thin layer of the low viscosity materials; that is, Flexitime Correct Flow was applied to the surface for identifying the roughened group, and Virtual Light Body was applied to the other specimens for identifying the polished group (Figure 2).

**Figure 3:**
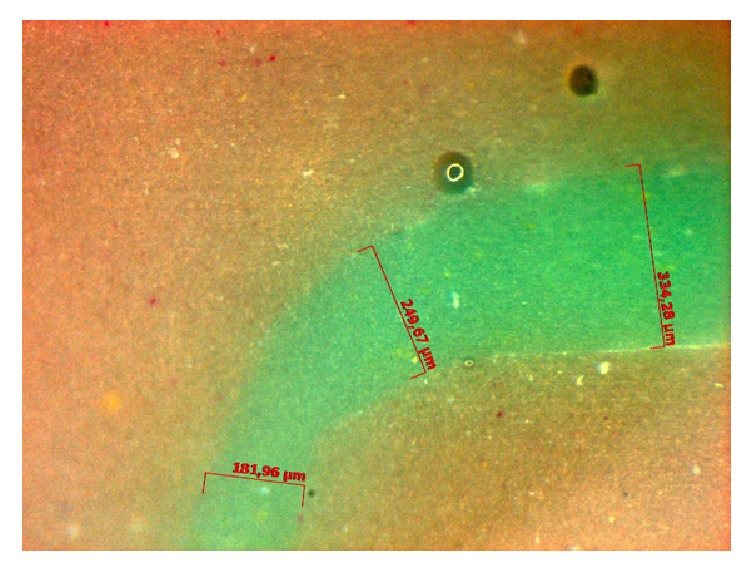
Film measurement held in stereomicroscope.

At the end of identification, plastic parts were separated, and the silicone film Flexitime Correct Flow was removed from the tooth by the stabilization silicone (Virtual, Ivoclar Vivadent). Subsequently, more low viscosity silicone was injected (Virtual, Ivoclar Vivadent) inside the film, and the assembly was poured and stabilized on a glass plate. After polymerization, the silicone film was sectioned in the buccolingual and mesiodistal directions, resulting in 4 pieces. For this purpose, #22 scalpel blades were used, which were replaced every five sections to maintain the pattern and not tear the film. 

(iv) Measurement of the film thickness: for each of the four segments—buccal, lingual, mesial, and distal—images of the cervical, axial, and occlusal areas were obtained. The images were obtained 24 hours after the manufacturing of the replicas, under microscope with a magnification of 70x. To measure the film thickness corresponding to the internal gap of the tooth/restoration interface, an image analysis software was used (Image Tool 3.0 for Windows, University of Texas, Health Science Center San Antonio, Texas, USA) (Figure 3).

For each section in the buccolingual direction 12 measurements were performed at different locations of the extent of film, and another 12 were made mesiodistally, in the same measurement sites. Thus, 24 measurements were performed for each crown. Three readings were made at each measurement site. The average of the readings was considered as the final value for each site.

**Figure 4:**
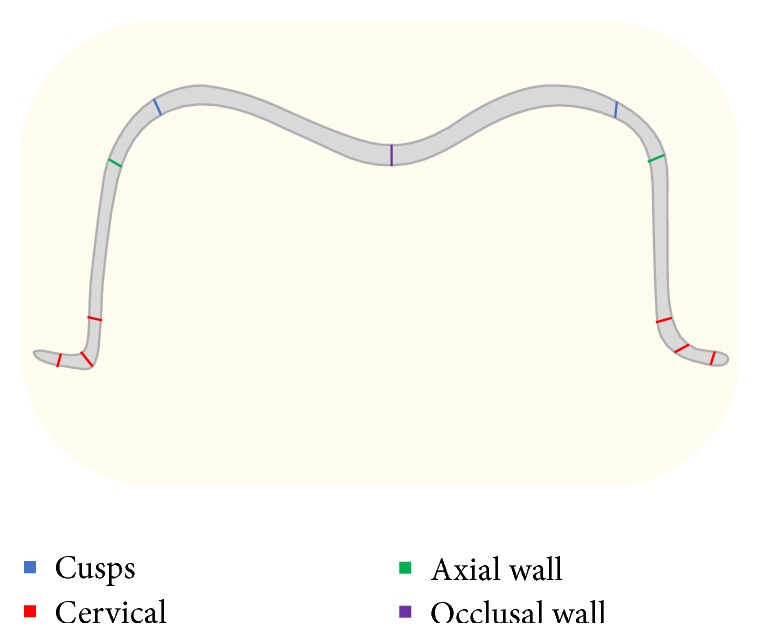
Schematic diagram of measurement areas.

The measurement points of the buccolingual and mesial-distal sections are listed as follows (Figure 4): (a) cervical wall; (b) cervicoaxial angle; (c) axial wall at the cervical third; (d) axial wall at the occlusal third; (e) axio occlusal angle; (f) occlusal wall.

In order to avoid confusion when performing the statistical test, the mean values obtained were plotted so that each region could be evaluated by comparison between groups. Thus, Group 1 was odd-numbered and Group 2 was even-numbered, so each number was correspondent to the region of the surfaces and evaluated within its group.

The statistical test used during this study was Shapiro-Wilk's and Mann–Whitney's at a significance level of 0,05%.

## 3. Results

Values were found in the Shapiro-Wilk's test of normality performed in both tested groups and their descriptive statistics. The Shapiro-Wilk's normality test indicated a nonuniform distribution of data. Because of this, they were treated statistically using a Mann–Whitney nonparametric test. Generally no significant differences were observed between both groups tested (*p* > 0.05), except for the region of the Cervical-Axial-Palatal Angle of G1 which presented higher value than G2 (*p* = 0.0494) (Table 1).

## 4. Discussion

The null hypothesis was accepted because, within the results found, one may observe that the statistical variation between both types of preparation finishing was virtually minimal. The only variable in measurement of the regions occurred in only one region and located only in the palatal region. The success of all-ceramic crowns is associated with several factors and laboratory steps that must be respected. Beyond aesthetics, a good marginal and internal adaptation are the ideal when implementing full crowns. This prevents any interference of the biofilm and possible marginal discoloration and caries recurrence among other factors [5]. If the restoration is not properly seated on the preparation it might generate the plaque accumulation and consequently trigger periodontal inflammation [22].

Obviously, further studies become necessary for us to extrapolate these scientific findings to clinical practice. But, within the results of this research, one might consider the possibility of clinical time saving during polishing of the preparations and the technical feasibility of crown preparation. Noticeably the acquisition device (Bluecam, Cerec) performed correctly in reading its function, from which it can be concluded that the polishing or not polishing preparation does not interfere with reading and computer-assisted design of all-ceramic crowns.

The instruments for evaluation and measurement of the marginal discrepancy can be optical microscopes [6, 13, 25], stereomicroscopes [2, 18], and scanning electron microscopes [7]. In this study, the choice for the stereomicroscope was defined due to the convenience of snapshots and reading and subsequent measurement of the surfaces in the same place beyond the sharpness of the images obtained. Another important feature was the ability of saving the image with the corresponding measurement value.

During the evaluation phase of the internal fit, no interference was found to be enough to require further adjustment; thus the ceramic restorations were crystallized without any sharp object contacting in addition to the milling cutters of the CAD/CAM, during the milling step. We believe that the CAD/CAM technology helps in manufacturing of ceramic crowns, because the steps that were previously made in the laboratory, taking days or weeks, now can be performed in a practical and easy way in less than 20 minutes for each crown (considering scanning, design, and milling).

Computer aided systems (CAD/CAM) are designed to simplify the processing of dental prostheses, producing restorations in less time and with equipment that enables an accurate reading [8]. Previously, low resolution scanners resulted in prostheses with poor marginal and internal fit fabricated with CAD/CAM systems. However, recent advances in technology, engineering, and materials brought CAD/CAM systems using high-precision scanners and more sophisticated software to scan complex shapes required in the office, such as that used in this research [8, 10].

In this study, the measurement of the internal fit reproduced previous studies. The choice for the internal replica technique was recommended by McLean and Von [26] because it is a nondestructive technique. In this technique, the film thickness can be captured for measurement, preserving the tooth/crown specimen [27]. The steps of image acquisition, design, and milling of restorations were standardized and performed by the same operator calibrated and trained in the use of this technology. Moreover, the ceramic material, lithium disilicate, the shade, and the artificial teeth were standardized, along with the PVS material used for simulation of the luting agent, in order to minimize weight of the metal device used for the simulation of the cement which was standardized during the setting of the PVS material.

One out of the 24 regions measured for each crown was found to be different between groups, in the region of axial-pulpal cervical angle. This led us to believe that during the roughening stage of restoration, which simulated the preparation unpolished, excessive pressure might have occurred. The preparations were sprinkled simulating the oral environment, so the tooth was placed on the mannequin. This variation may have occurred due to a slight difference in the angle of the diamond point during the surface roughening procedure.

A recently published study that evaluated the marginal and internal fit, of two different milling systems, where a system used selective laser melting and the other a CAD/CAM system, found that the first system presented superior results for marginal and internal fit [28]. Maybe it would be interesting to investigate the polishing of the preparation and increase the number of groups to be evaluated, such as using the laser system mentioned above.

Despite the limitations of this study it can be concluded that the full crowns manufactured with CAD/CAM technology on preparations with or without polishing showed no statistically significant differences in the evaluation of marginal adaptation. Also it can be concluded that polishing preparations of full crowns fabricated with CAD/CAM technology are optional. This may be waived by the dentist reducing the clinical time during the preparation procedure. However it would be interesting to carry out further research evaluating these parameters on natural teeth.

## Figures and Tables

**Table 1:** Median, minimum and maximum values of the G1 and G2.

Measured area (G1)	*Median, minimum, and maximum values*	Measured area (G2)	*Median, minimum, and maximum values*
1	119.6000 (86.0300–192.3400)	1	155.7600 (70.5000–340.8300)
2	173.0250 (84.9700–356.7100)	2	117.5150 (78.9000–489.8000)
3	126.5500 (80.9200–489.8000)	3	122.1350 (96.7400–345.7600)
4	139.5150 (57.3100–217.5800)	4	148.5250 (40.9800–209.8000)
5	128.1900 (77.0990–189.3400)	5	123.2050 (69.7400–380.9200)
6	145.9850 (30.9800–295.2700)	6	125.3350 (63.6500–374.4100)
7	193.5450 (78.3300–291.2800)^*∗*^	7	100.8250 (76.3000–486.4000)^*∗*^
8	119.3050 (52.0900–220.3600)	8	85.1150 (38.71000–205.3000)
9	143.0700 (54.9200–217.6800)	9	137.6200 (64.7200–239.7000)
10	109.7900 (85.0600–236.3700)	10	96.2800 (56.8000–306.1300)
11	128.3800 (97.5000–309.7700)	11	112.6450 (65.1200–234.4200)
12	169.8800 (66.2000–311.5200)	12	125.6650 (56.1900–607.4400)
13	154.3450 (54.0300–307.9800)	13	186.6650 (112.5600–422.8300)
14	103.2150 (44.2000–261.27000)	14	153.3550 (41.3800–369.6500)
15	185.6550 (66.2100–309.5600)	15	130.6959 (44.3000–506.6800)
16	191.7900 (77.9800–379.3400)	16	179.5100 (68.8400–416.9000)
17	229.5000 (84.2100–526.8800)	17	212.9400 (110.7100–268.3500)
18	272.7150 (76.3500–322.3800)	18	215.8650 (125.7500–409.3900)
19	204.7700 (64.5700–455.7100)	19	189.9250 (52.6900–373.64000)
20	241.3250 (184.7400–351.1400)	20	210.3200 (149.7300–534.4600)
21	211.7150 (108.9100–318.0700)	21	254.7500 (87.7900–644.8800)
22	267.4700 (159.0800–397.1200)	22	237.4450 (151.6300–427.7400)
23	278.8050 (116.5800–491.3800)	23	191.2800 (95.0500–346.4500)
24	305.3850 (130.5000–355.2700)	24	216.2850 (161.5000–409.2100)

^*∗*^Evaluated parameters showed statistically significant difference with a value of *p* = 0.0494. This difference reflects the comparison of cervical-axial-palatal angle between G1 and G2. The other areas analyzed did not present a statistically significant difference with *p* value > 0.05.
